# Reduced Plasma Cardiac Myosin-Binding Protein-C Concentrations in Cats with Hypertrophic Cardiomyopathy: Evidence from Chronic, Treated Disease

**DOI:** 10.3390/ani16193084

**Published:** 2026-09-30

**Authors:** Nutcha Tanakwang, Natcha Sakunasing, Sarawut Kumphune, Raktham Mektrirat, Kannika Na Lampang, Chavalit Boonyapakorn, Wanpitak Pongkan

**Affiliations:** 1 Faculty of Veterinary Medicine, Chiang Mai University, Chiang Mai 50100, Thailand; nutcha_tana@cmu.ac.th (N.T.); natcha_sakunasing@cmu.ac.th (N.S.); raktham.m@cmu.ac.th (R.M.); kannika.nalampang@cmu.ac.th (K.N.L.); chavalit.b@cmu.ac.th (C.B.); 2 Cardiopulmonary Clinic, Northern Regional Veterinary Healthcare and Learning Center, Faculty of Veterinary Medicine, Chiang Mai University, Chiang Mai 50100, Thailand; 3 Biomedical Engineering Institute (BMEI), Chiang Mai University, Chiang Mai 50100, Thailand; sarawut.kumphune@cmu.ac.th; 4 Biomedical Engineering and Innovation Research Center, Chiang Mai University, Chiang Mai 50100, Thailand

**Keywords:** cardiac myosin-binding protein C, feline hypertrophic cardiomyopathy, heart failure, N-terminal pro-B-type natriuretic peptide

## Abstract

Hypertrophic cardiomyopathy (HCM), the most common cardiac disease in cats, is associated with genetic variants, including *MYBPC3*, which encodes cardiac myosin-binding protein C (cMyBP-C), a key sarcomeric protein involved in myocardial contractility. Although circulating cMyBP-C has emerged as a biomarker of myocardial injury in humans, its clinical relevance in feline HCM remains unclear. This study characterized plasma cMyBP-C and N-terminal pro-B-type natriuretic peptide (NT-proBNP) concentrations across different stages of feline HCM, evaluated their diagnostic performance, and assessed their associations with echocardiographic parameters. A cross-sectional study included 65 cats: 25 healthy cats, 25 with subclinical HCM, and 15 with clinical HCM (chronic and treated). Plasma NT-proBNP was significantly higher in clinical HCM than in healthy cats (*p* = 0.0117), whereas plasma cMyBP-C was lower in clinical HCM than in subclinical HCM (*p* = 0.0462) and healthy cats (*p* = 0.001). At a cut-off of <1.270 ng/mL, plasma cMyBP-C showed 76.19% sensitivity and 65.38% specificity for distinguishing HCM. NT-proBNP alone showed higher overall diagnostic accuracy (76.67%) than the combined use of cMyBP-C and NT-proBNP. Neither plasma NT-proBNP nor cMyBP-C correlated significantly with echocardiographic parameters. Reduced circulating cMyBP-C concentrations in cats with chronic, treated HCM suggest a biomarker pattern distinct from that reported in acute myocardial injury and warrant further investigation.

## Simple Summary

Hypertrophic cardiomyopathy is the most common heart disease in cats and can be associated with changes in genes, including *MYBPC3*, a gene that provides instructions for making cardiac myosin-binding protein C (cMyBP-C), a protein important for normal heart muscle function. Changes in cMyBP-C have been studied as an indicator of heart muscle injury in humans, but its role in feline heart disease is not well understood. This study investigated circulating cMyBP-C concentrations in healthy cats and cats with different stages of hypertrophic cardiomyopathy to determine its potential value in assessing heart disease. Cats with chronic, treated hypertrophic cardiomyopathy had lower cMyBP-C concentrations than healthy cats and cats with subclinical disease. This finding differs from the increased cMyBP-C levels reported in sudden heart muscle injury. Although cMyBP-C showed potential as an additional indicator of heart disease, it was less accurate than N-terminal pro-B-type natriuretic peptide (NT-proBNP), a blood test currently used to assess heart problems in cats. These findings suggest that cMyBP-C may provide complementary information but cannot replace existing tests at present. Further studies are needed to understand the biological role of cMyBP-C in feline heart disease.

## 1. Introduction

Cardiomyopathies are the most common form of heart disease in domestic cats [1], with hypertrophic cardiomyopathy (HCM) being the most prevalent myocardial disorder [2,3]. HCM is characterized by diffuse or regional increases in left ventricular (LV) wall thickness in the absence of LV dilation [4] and may occur as a primary disease or secondary to systemic conditions such as hypertension, hyperthyroidism, and hypersomatotropism [5,6,7,8,9]. Primary HCM has been associated with genetic variants in sarcomeric proteins [10,11], including cardiac myosin-binding protein C (cMyBP-C), encoded by *MYBPC3* [12,13]. However, the genetic basis remains unknown in most affected cats, and the association between reported genetic variants and HCM is not consistent across feline populations [14,15,16,17,18]. Cats with HCM may remain asymptomatic for prolonged periods [19,20] but can develop life-threatening complications, including heart failure (HF), arterial thromboembolism (ATE), and sudden death [21]. Therefore, circulating biomarkers that may facilitate the detection and characterization of HCM could have clinical value in affected cats.

cMyBP-C is a specific cardiac sarcomere thick filament protein that regulates heart muscle contractility by binding its N-terminal domains to actin-myosin through dynamic phosphorylation [22,23,24]. Several previous studies have demonstrated that alterations in the phosphorylation process of cMyBP-C are associated with impaired actomyosin cross-bridge formation, ultimately leading to contractile dysfunction and the development of HF [25,26,27,28]. When cardiomyocytes are injured, cMyBP-C can be released into the bloodstream [29]. Similar to cardiac troponin and other cardiomyocyte-specific proteins, circulating cMyBP-C may reflect cardiac injury [22]. Studies in experimental animal models and humans have shown that myocardial injury associated with myocardial infarction (MI) and ischemia can result in elevated plasma cMyBP-C concentrations [29,30,31,32,33,34]. In addition, previous studies have demonstrated that circulating cMyBP-C concentrations are associated with HF severity [25,33]. Consequently, circulating cMyBP-C has emerged as a promising biomarker of myocardial injury in human medicine because of its rapid release kinetics, which enable earlier detection than traditional cardiac troponins, as well as its potential prognostic value in patients with HF [30,32,35,36]. In contrast, studies in human and animal models of HCM have demonstrated reduced tissue cMyBP-C expression, which has been associated with *MYBPC3*-related alterations [37,38]. These findings emphasize the distinct biological implications of tissue and circulating cMyBP-C measurements. While myocardial cMyBP-C reflects sarcomeric protein abundance, circulating cMyBP-C may provide information regarding cardiomyocyte damage and protein release into the bloodstream. However, evidence regarding circulating cMyBP-C in veterinary medicine remains scarce, particularly in cats, and its clinical significance in feline HCM has not been fully established.

N-terminal pro-B-type natriuretic peptide (NT-proBNP) is a widely used circulating cardiac biomarker in feline cardiology for detecting cardiac disease and assessing disease severity [39,40]. Although NT-proBNP is not specific for HCM, its established clinical use provides a relevant comparator for evaluating the potential diagnostic utility of circulating cMyBP-C. Therefore, evaluating plasma cMyBP-C concentrations across different stages of feline HCM may provide insights into its potential role as a diagnostic and disease-stage biomarker in affected cats. The objective of this study was to (1) compare the plasma levels of cMyBP-C and NT-proBNP in cats at various stages of HCM; (2) assess the diagnostic accuracy of plasma cMyBP-C in identifying HCM in feline patients; and (3) evaluate the associations between plasma levels of cMyBP-C and echocardiographic parameters related to disease severity in HCM cats. We hypothesized that (i) plasma cMyBP-C concentrations would differ among healthy cats and cats with subclinical (Sub-C) and clinical HCM; (ii) the diagnostic performance of plasma cMyBP-C would be similar to that of NT-proBNP for diagnosing HCM in cats; and (iii) plasma cMyBP-C concentrations would be associated with echocardiographic parameters of HCM severity.

## 2. Materials and Methods

### 2.1. Animal Model and Research Protocol

**Figure 1:**
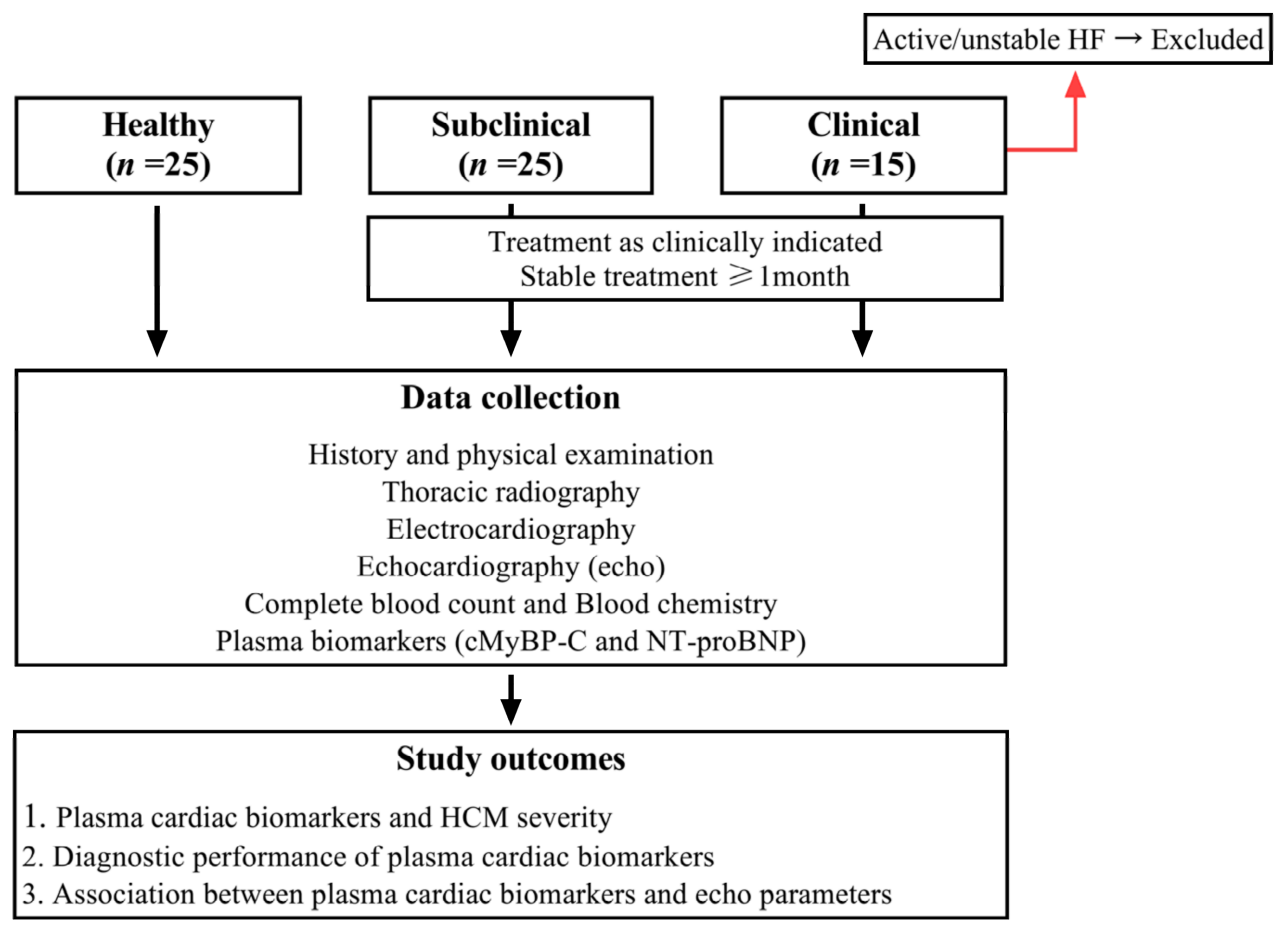
Schematic representation of the study protocol involving 25 healthy cats and 40 cats with hypertrophic cardiomyopathy (HCM). Clinical HCM cats with active or unstable heart failure (HF) were excluded (red arrow). cardiac myosin-binding protein C (cMyBP-C); N-terminal pro-B-type natriuretic peptide (NT-proBNP).

This cross-sectional study included 65 cats with no restrictions on age, sex, or breed. Cats were recruited from those presented to a small animal hospital for routine health examinations, castration, or ovariohysterectomy, as well as cats diagnosed with an HCM phenotype by a veterinary cardiologist. The cats were then divided into three groups based on the American College of Veterinary Internal Medicine (ACVIM) classification and HCM consensus guidelines [4]: healthy (Stage A, *n* = 25), subclinical HCM (Sub-C; Stages B1 and B2 combined, *n* = 25), and clinical HCM (Stages C and D combined, *n* = 15). HCM was diagnosed by echocardiography based primarily on LV wall thickness, with LA size and other echocardiographic findings considered as part of the overall cardiac assessment. Clinical HCM was diagnosed by a veterinary cardiologist based on clinical signs consistent with HF, including dyspnea, tachypnea, lethargy, and decreased appetite, in conjunction with thoracic radiographic evidence of pulmonary edema, pleural effusion, or cardiomegaly and echocardiographic findings consistent with HCM. Cats with inflammatory or infectious diseases, respiratory diseases, malignant tumors, neurological disorders, congenital heart disease, or acute or unstable HF were excluded. Cats with subclinical or clinical HCM were managed according to the ACVIM consensus recommendations [4]. Medical treatment was prescribed when clinically indicated. Cats receiving medications had been on a stable treatment regimen for at least 1 month before enrollment, and all medications were recorded. All cats underwent demographic data collection, physical examination, thoracic radiography, electrocardiography (ECG), echocardiography, and blood collection for complete blood count (CBC), blood chemistry (BC) panel (including blood urea nitrogen, creatinine, alanine transaminase, alkaline phosphatase, total protein, and albumin), and cardiac biomarker measurements (cMyBP-C and NT-proBNP), as summarized in Figure 1. Written informed consent was obtained from all owners before enrollment.

### 2.2. Physical Examination and Thoracic Radiography

Physical examination was performed as part of the routine clinical assessment and included body condition score (9-point scale), heart rate (beats per minute; bpm), respiratory rate (times per minute; tpm), and rectal temperature (°F). Thoracic radiographs were obtained in right lateral and dorsoventral projections. Cardiomegaly was assessed using the vertebral heart score (VHS), while left atrial (LA) size was evaluated using the vertebral left atrial score (VLAS), modified vertebral left atrial score (M-VLAS), and radiographic left atrial dimension (RLAD). VLAS was measured according to a method previously described in feline patients [41], whereas M-VLAS and RLAD were adapted from methods originally developed and evaluated in canine studies [42].

### 2.3. ECG Recordings

Electrocardiography was performed as part of the comprehensive cardiac evaluation protocol to assess cardiac electrical activity and identify potential rhythm abnormalities in all enrolled cats. A standard six-lead ECG was performed using three bipolar limb leads (I, II, and III) and three augmented unipolar limb leads (aVR, aVL, and aVF). ECG was recorded for 2 min at a paper speed of 50 mm/s and a sensitivity of 20 mm/mV. Recordings were obtained without sedation in gently restrained cats positioned in right lateral recumbency, with limb electrodes placed according to the standard technique [43]. The evaluated ECG parameters included heart rate, P-wave amplitude (mV), P-wave duration (s), PR interval (s), R-wave amplitude (mV), QRS duration (s), ST-segment amplitude (mV), T-wave amplitude (mV), QT interval (s), and mean electrical axis (MEA; degrees).

### 2.4. Echocardiographic Examination

Echocardiographic examinations were performed using an ultrasound system (CX50 xMatrix, Philips, Bothell, WA, USA) with two-dimensional (2D), M-mode, and Doppler techniques [4,44]. All examinations and measurements were performed by a single experienced veterinary cardiologist without sedation in gently restrained cats. Standard echocardiographic views were investigated by a single experienced veterinary cardiologist in the right lateral recumbency (right parasternal long and short views) and left lateral recumbency (left apical four-chamber view).

Left atrial size was assessed by two-dimensional echocardiography. The LA and aortic (Ao) dimensions were measured at early diastole from the right parasternal short-axis view using the inner-edge-to-inner-edge method, and the LA-to-aortic ratio (LA/Ao) was calculated. An LA/Ao ratio <1.5 was considered indicative of normal LA size based on previously established criteria [45].

Left ventricular (LV) dimensions and wall thickness were measured using 2D-guided M-mode echocardiography from the right parasternal short-axis view, including left ventricular posterior wall thickness at end-diastole (LVPWd), LV internal dimension at end-diastole (LVIDd), interventricular septal thickness at end-diastole (IVSd), left ventricular posterior wall thickness at end-systole (LVPWs), LV internal dimension at end-systole (LVIDs), and interventricular septal thickness at end-systole (IVSs). LVPWd and IVSd were measured at end-diastole using the leading-edge-to-leading-edge technique. Measurements were obtained over three consecutive cardiac cycles and averaged for analysis [46]. LV hypertrophy was defined as a wall thickness of ≥6 mm in either LVPWd or IVSd [4]. HCM classification was based on individual echocardiographic measurements rather than group mean values. Echocardiographic assessments were performed with access to relevant clinical information but blinded to biomarker results. Papillary muscle hypertrophy was identified when the combined papillary muscle area was >0.8 cm^2^ [47] and was considered as part of the overall echocardiographic assessment. However, papillary muscle area was not included as an independent parameter in the statistical analysis. Secondary causes of LV hypertrophy could not be completely excluded.

Systolic function was assessed using fractional shortening (FS), ejection fraction (EF), stroke volume (SV), and cardiac output (CO), with EF and SV derived using the Teichholz method [48]. Moreover, the Doppler mode was used to evaluate cardiac diastolic function [49] by measuring the peak velocity blood flow from LV relaxation in early diastole (MV E peak velocity) and the ratio of peak velocity blood flow from LV relaxation in early diastole to peak velocity blood flow in late diastole caused by atrial contraction (MV E/A).

Cats with HCM were staged according to the ACVIM consensus guidelines [4]. Stage B1 included asymptomatic cats at low risk of imminent HF or ATE, whereas Stage B2 included asymptomatic cats with increased risk, defined by severe LA enlargement (LA/Ao ≥ 1.8) [1] and/or additional risk factors, including spontaneous echocardiographic contrast, intracardiac thrombus, arrhythmias, or left ventricular systolic dysfunction.

### 2.5. Measurement of Plasma cMyBP-C and NT-proBNP

Three milliliters of blood were collected into heparinized tubes and centrifuged at 2000 rpm for 20 min at 2–8 °C. Plasma was transferred to cryovials and stored at −80 °C until analysis. Plasma cMyBP-C and NT-proBNP concentrations were measured using a commercially available sandwich enzyme-linked immunosorbent assay (ELISA). Plasma samples were stored at −80 °C for approximately 2 months before analysis and underwent a single freeze–thaw cycle.

Plasma cMyBP-C was measured using a feline cMyBP-C double-antibody sandwich ELISA kit (Wuhan Feiyue Biotechnology Company, Wuhan, China) according to the manufacturer’s instructions. According to the manufacturer, the assay had a sensitivity of 0.1 ng/mL, a detection range of 1–16 ng/mL, and a coefficient of variation <9%, with no significant cross-reactivity or interference with *MYBPC3* analogues. Samples and standards were assayed in duplicate. Samples were diluted according to the manufacturer’s protocol, incubated with HRP-conjugated reagent for 60 min at 37 °C, washed five times, and incubated with substrate for 15 min at 37 °C in the dark. After the addition of the stop solution, optical density was measured at 450 nm using a microplate reader. Samples were analyzed using two ELISA plates, with samples from healthy, subclinical HCM, and clinical HCM cats distributed across both plates to minimize potential inter-plate variability.

Plasma NT-proBNP was measured using a feline NT-proBNP sandwich ELISA kit (MyBioSource, Inc., San Diego, CA, USA) according to the manufacturer’s instructions. Samples were incubated with anti-NT-proBNP antibody and streptavidin-HRP for 60 min at 37 °C, washed five times, and incubated with substrate for 10 min at 37 °C in the dark. After the addition of the stop solution, optical density was measured at 450 nm using a microplate reader.

### 2.6. Statistical Analysis

Statistical analysis was performed using the GraphPad Prism software version 9.0.0. For qualitative data, comparisons between two groups and more were made using the chi-square test. The Shapiro–Wilk test was used to test the distribution of quantitative data. The normally distributed data are presented as mean ± standard deviation (SD), whereas the nonnormally distributed data are presented as median and interquartile range (IQR). Continuous variables were assessed for normality before statistical analysis. One-way ANOVA was used to compare the means of more than two groups of parametric data, followed by Tukey’s post hoc test when significant differences were identified. The Kruskal–Wallis test was used for nonparametric data, followed by Dunn’s post hoc test for pairwise comparisons. The receiver operating characteristic (ROC) curve analysis was used to calculate the optimum cut-off values of plasma cMyBP-C and NT-proBNP levels, using the echocardiographic results as the gold standard. Cross-tabulation was performed to determine the sensitivity, specificity, positive predictive value (PPV), negative predictive value (NPV), and accuracy of each test. The echocardiographic results were considered the gold standard for this comparison. Correlation analyses were performed to investigate the relationships between plasma cardiac biomarkers and echocardiographic parameters in HCM cats. Pearson’s correlation coefficient was used for normally distributed variables, whereas Spearman’s rank correlation test was used when at least one variable showed a non-normal distribution. A *p*-value of <0.05 was considered statistically significant.

## 3. Results

### 3.1. Breed Distribution

**Figure 2:**
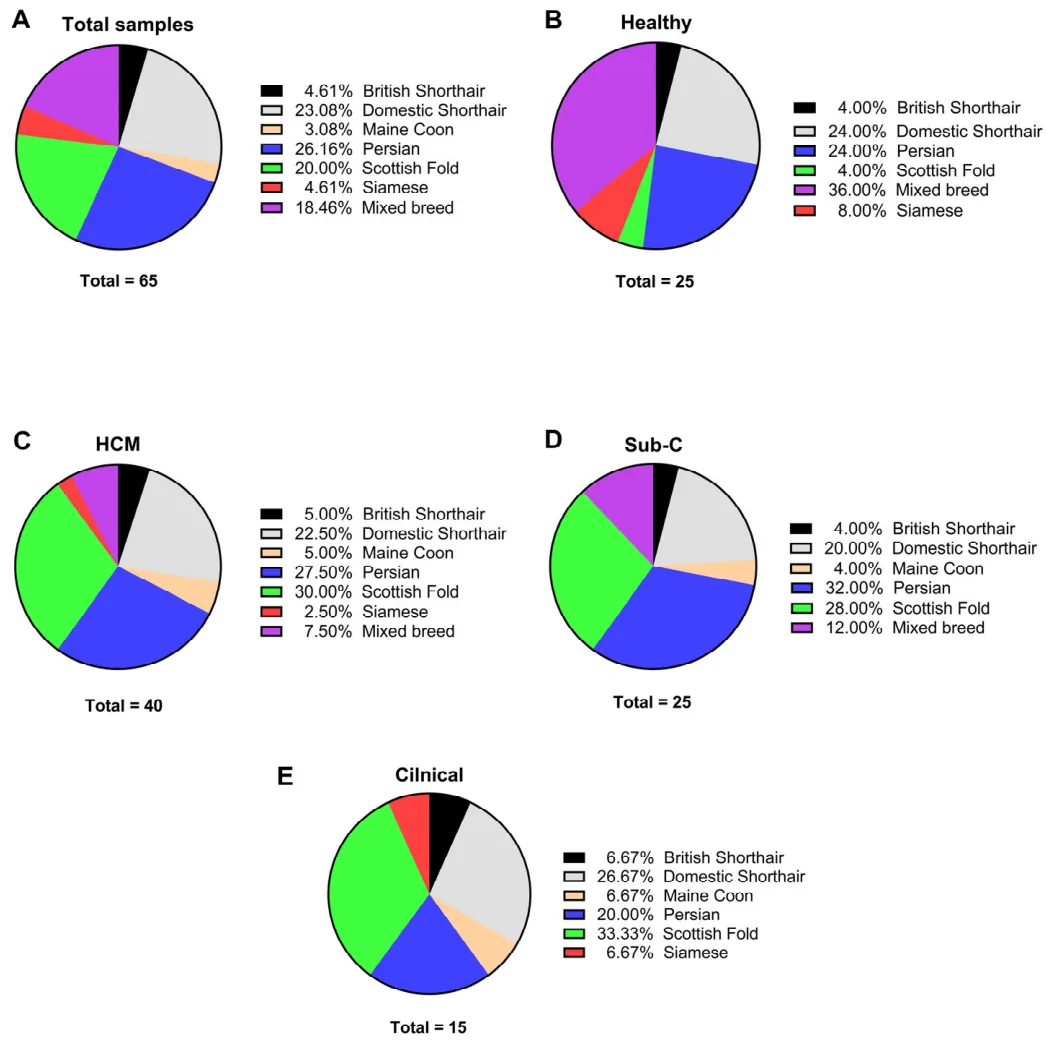
Pie chart showing the proportion of breed distribution in each category. Total samples (**A**), healthy cats (**B**), hypertrophic cardiomyopathy (HCM) cats (**C**), subclinical HCM (Sub-C) (**D**), and clinical HCM cats (**E**). Legend colors are consistent across all panels: black, British Shorthair; gray, Domestic Shorthair; beige, Maine Coon; blue, Persian; green, Scottish Fold; red, Siamese; and purple, mixed breed. The total number of cats in each category is indicated below each pie chart.

The study included 65 cats from 7 breeds: British Shorthair (*n* = 3), Domestic Shorthair (*n* = 15), Maine Coon (*n* = 2), Persian (*n* = 17), Scottish Fold (*n* = 13), Siamese (*n* = 3), and Mixed breed (*n* = 12) (Figure 2A). Of these, 25 cats were healthy, and 40 cats were diagnosed with HCM by cardiologists. Among the HCM cats, Scottish Fold and Persian were the most frequently represented breeds, comprising 30.0% and 27.5% of the affected cats, respectively, followed by Domestic Shorthair (22.5%), Mixed breed (7.5%), British Shorthair (5.0%), Maine Coon (5.0%), and Siamese (2.5%) (Figure 2C). The breed distributions in healthy, Sub-C, and clinical HCM cats are shown in Figure 2B,D,E, respectively.

### 3.2. Current Medications

Medical treatment was prescribed according to clinical indications and current consensus recommendations [4,10]. The medication record showed that cats in the Sub-C stage received angiotensin-converting enzyme inhibitors (ACEis) (*n* = 3), antiplatelet drugs (*n* = 3), β-blocker (*n* = 2), and calcium channel blockers (CCBs) (*n* = 2). Cats in the clinical stage received ACEis (*n* = 6), antiplatelet drugs (*n* = 15), β-blocker (*n =* 4), CCBs (*n* = 2), furosemide (*n* = 10), pimobendan (*n* = 3), and spironolactone (*n* = 3). Detailed information on medication type, dose, concurrent medication use, and overall treatment duration is provided in Table S1.

### 3.3. Signalment and Physical Examination

The Sub-C group included Stage B1 (*n* = 12) and Stage B2 (*n* = 13). The clinical group included 14 cats with Stage C and 1 with Stage D. Post-hoc pairwise comparisons showed that heart rate was higher in the clinical and Sub-C groups compared to the healthy group (*p* = 0.0442 and *p* = 0.0305, respectively), while rectal temperature was significantly lower in the clinical group compared with the Sub-C (*p* = 0.0385) and healthy groups (*p* = 0.0018). However, there were no statistical differences among the groups regarding sex, age, body weight, BCS, and respiratory rate (Table 1).

### 3.4. Complete Blood Count and Blood Biochemistry

Post hoc pairwise comparisons showed that BUN was significantly higher in the clinical and Sub-C HCM groups than in the healthy group (*p* = 0.0002 and *p* = 0.0117, respectively), while creatinine concentration was significantly higher in the clinical HCM group compared with the healthy group (*p* = 0.0232). Other BC profiles, except total protein, remained within the normal range, with no significant differences between the groups. Similarly, the CBC was also within normal limits, with no significant differences between the groups (Table 2).

### 3.5. Electrocardiographic Findings

No cardiac arrhythmias, including ventricular premature complexes or atrial fibrillation, were identified in any enrolled cats. There were no significant differences in ECG morphology and MEA measurements between the healthy, Sub-C, and clinical groups. However, post hoc pairwise comparisons showed that heart rate was significantly higher in the clinical and Sub-C HCM groups compared with the healthy group (*p* = 0.004 and *p* = 0.042, respectively). Nevertheless, all ECG parameters in both the Sub-C and clinical groups were within normal limits.

### 3.6. Thoracic Radiographic Findings

Post hoc pairwise comparisons showed that the VHS was significantly higher in the clinical HCM group compared with the Sub-C (*p* = 0.0004) and healthy groups (*p* < 0.0001). Thoracic radiography evaluated the LA size using three methods: VLAS, M-VLAS, and RLAD. The clinical HCM group had significantly higher values than the Sub-C and healthy groups for VLAS (*p* = 0.0012 and *p* = 0.0053), M-VLAS (*p* = 0.0001 and *p* < 0.0001), and RLAD (*p* = 0.0150 and *p* < 0.0001). Additionally, RLAD values were significantly higher in the subclinical HCM group compared with the healthy group (*p* = 0.0051) (Table 3).

### 3.7. Echocardiographic Finding

Post hoc pairwise comparisons showed that the LA/Ao ratio was significantly higher in both clinical and Sub-C cats compared with healthy cats (*p* < 0.0001 and *p* < 0.0001, respectively). The LA/Ao ratio was also significantly higher in clinical HCM cats compared with Sub-C cats (*p* = 0.0190). In addition, LA diameter was significantly larger in clinical HCM cats than in Sub-C and healthy cats (*p* = 0.0292 and *p* < 0.0001, respectively).

Left ventricular wall thickness parameters during diastole, including IVSd and LVPWd, were significantly higher in clinical and Sub-C cats compared with healthy cats (IVSd: *p* < 0.0001 and *p* < 0.0001; LVPWd: *p* < 0.0001 and *p* = 0.0024, respectively). Furthermore, LVPWd was significantly higher in clinical HCM cats compared with Sub-C cats (*p* = 0.0002). LVIDd was significantly lower in clinical HCM cats compared with healthy cats (*p* = 0.0233). During systole, IVSs and LVPWs were significantly higher in clinical HCM cats compared with Sub-C and healthy cats (IVSs: *p* = 0.0027 and *p* < 0.0001; LVPWs: *p* = 0.0024 and *p* < 0.0001, respectively). In addition, IVSs were significantly higher in subclinical HCM cats compared with healthy cats (*p* = 0.0003). Mitral valve E-wave velocity was significantly higher in clinical HCM cats compared with Sub-C and healthy cats (*p* = 0.0067 and *p* = 0.0079, respectively). The E/A ratio differed significantly between Sub-C and clinical HCM cats (*p* = 0.0075). No significant differences were observed among groups for systolic function parameters, including %FS, %EF, SV, and cardiac output (Table 3).

### 3.8. Plasma cMyBP-C Levels and Plasma NT-proBNP Levels

**Figure 3:**
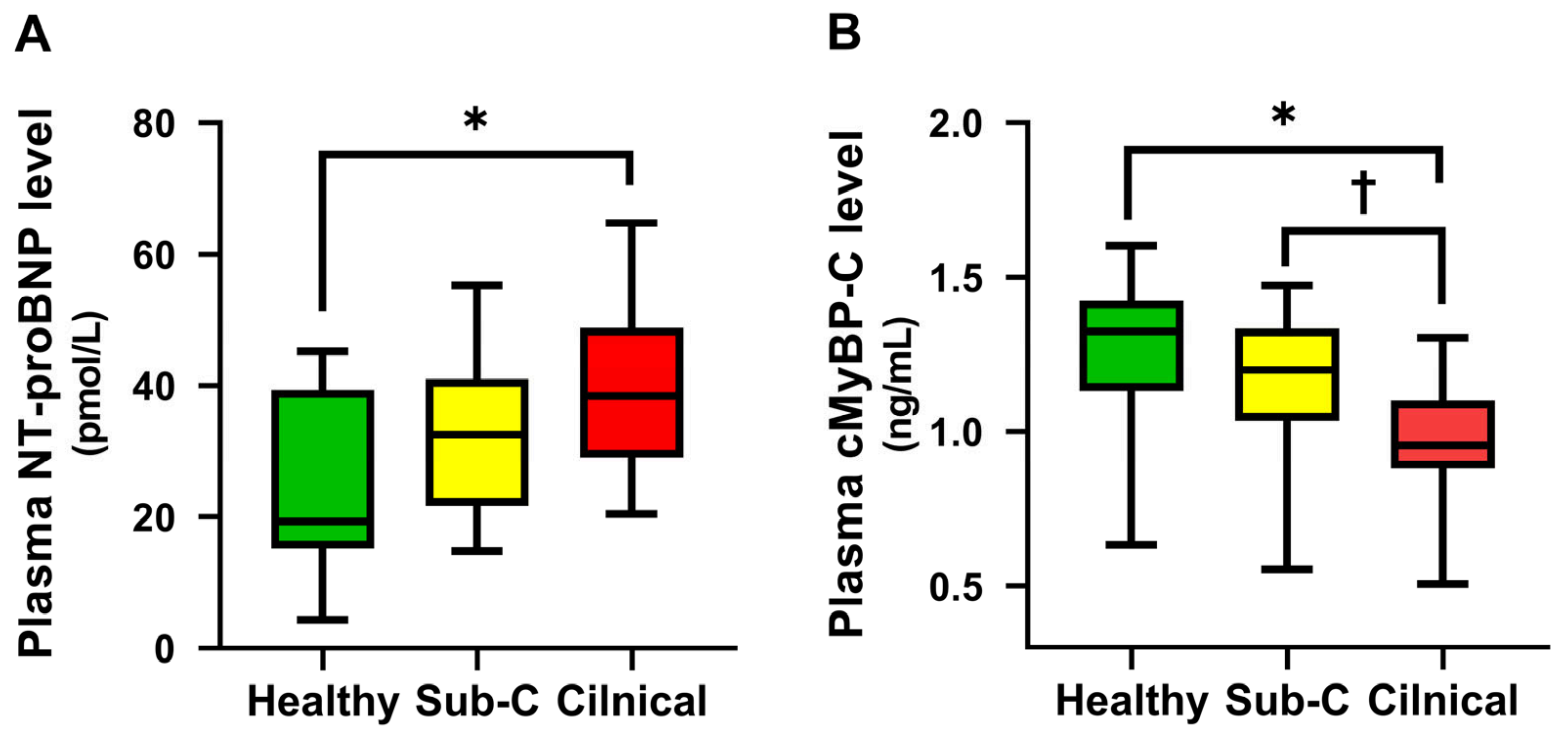
Plasma N-terminal pro-B-type natriuretic peptide (NT-proBNP) and cardiac myosin-binding protein C (cMyBP-C) levels in healthy and hypertrophic cardiomyopathy (HCM) cats (*n* = 65): (**A**) The median plasma NT-proBNP levels were significantly higher in clinical HCM cats (red box) compared to healthy cats (green box). (**B**) The median plasma cMyBP-C level was significantly lower in the clinical group compared to the Sub-C (yellow box) and healthy groups. Boxes = interquartile ranges; whiskers = minimum and maximum. * *p* < 0.05 vs. healthy group, ^†^ *p* < 0.05 vs. subclinical HCM (Sub-C) group.

The plasma NT-proBNP levels of the clinical HCM group (39.40 ± 13.30 pmol/L) were higher than those of the healthy group (19.29 [15.21–39.35] pmol/L, *p* = 0.0117), but there was no significant difference when compared with the Sub-C group (32.45 ± 11.89 pmol/L, *p* = 0.6899) (Figure 3A). In contrast, the plasma cMyBP-C levels were lower in the clinical group (0.977 ± 0.197 ng/mL) compared to the Sub-C (1.162 ± 0.242 ng/mL, *p* = 0.0462) and the healthy group (1.324 [1.132–1.423] ng/mL, *p* = 0.001) (Figure 3B).

### 3.9. Diagnostic Performance of Plasma cMyBP-C for Diagnosing HCM in Cats

**Figure 4:**
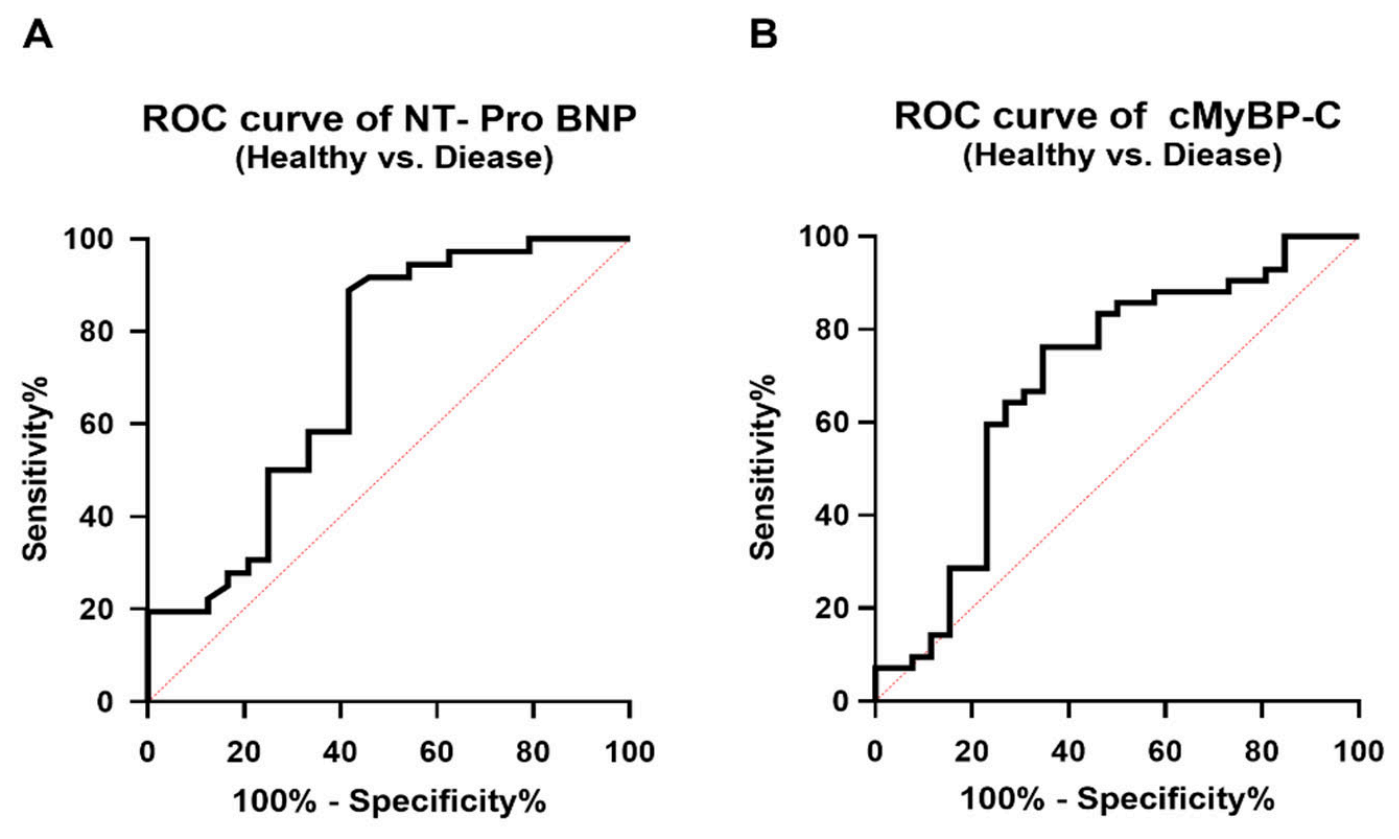
Receiver operating characteristic (ROC) curve of plasma cardiac biomarkers for the diagnosis of feline hypertrophic cardiomyopathy (HCM): (**A**) Plasma N-terminal pro-B-type natriuretic peptide (NT-proBNP) at a cut-off of >20.64 pmol/L (area under the curve [AUC] = 0.7118, sensitivity = 88.89%, specificity = 58.33%). (**B**) Plasma cardiac myosin-binding protein C (cMyBP-C) at a cut-off of <1.270 ng/mL (AUC = 0.6905, sensitivity = 76.19%, specificity = 65.38%).

ROC curve analysis was performed to evaluate the diagnostic performance of plasma NT-proBNP and cMyBP-C levels for discriminating between healthy cats and HCM cats. Plasma NT-proBNP showed an area under the curve (AUC) of 0.7118 (95% CI: 0.5693–0.8543), with a cut-off value of >20.64 pmol/L, sensitivity of 88.89%, and specificity of 58.33% (Figure 4A). Plasma cMyBP-C showed an AUC of 0.6905 (95% CI: 0.5519–0.8291), with a cut-off value of <1.270 ng/mL, sensitivity of 76.19%, and specificity of 65.38% (Figure 4B). For combined biomarker analysis, application of the “OR” rule resulted in sensitivities exceeding 90%. The highest sensitivity was observed with serial testing starting with NT-proBNP (97.22%). Application of the “AND” rule resulted in specificities and positive predictive values (PPV) exceeding 90%, with lower sensitivities. The lowest sensitivity was observed for serial testing starting with cMyBP-C (48.65%). Diagnostic performance parameters for combined testing strategies are summarized in Table 4.

### 3.10. Correlation Analysis

Correlation analysis revealed that neither plasma NT-proBNP nor plasma cMyBP-C concentrations were significantly associated with any echocardiographic parameters, including left atrial size (LA/Ao ratio), left ventricular structural parameters (IVSd, LVIDd, LVPWd, and LVIDs), systolic function parameters (EF, FS, SV, and CO), or diastolic function parameters (MV E velocity and E/A ratio) (Table 5).

## 4. Discussion

This study investigated the plasma levels of cMyBP-C and NT-proBNP in cats with various stages of feline HCM in comparison to healthy cats and assessed the correlation between plasma cMyBP-C and NT-proBNP and echocardiographic parameters. The first major finding was a distinct biomarker profile in cats with chronic, treated clinical HCM, characterized by higher NT-proBNP and lower cMyBP-C concentrations. Second, circulating cMyBP-C concentrations demonstrated modest diagnostic performance for distinguishing cats with HCM from healthy cats, whereas NT-proBNP showed better diagnostic performance. Third, neither plasma cMyBP-C nor NT-proBNP concentrations showed significant correlations with echocardiographic parameters in this study.

HCM is the most common cardiac condition in cats, accounting for approximately 60% of feline cardiomyopathy cases diagnosed by echocardiography [50]. It has been reported across various breeds, including both mixed-breed and purebred cats [4,51]. In our study population, however, Scottish Fold and Persian cats accounted for the largest proportion of the HCM group. Echocardiographic and thoracic radiographic findings were consistent with cardiac remodeling in HCM, including concentric LV hypertrophy, LA enlargement, and altered diastolic filling in more advanced disease [52,53,54]. Clinical HCM cats also exhibited higher heart rates and lower rectal temperatures, which may reflect compensatory physiological responses to impaired cardiac performance, consistent with a previous study [55]. Collectively, these findings reflected structural and clinical differences among the study groups and were consistent with HCM severity according to ACVIM staging [4].

Our first major finding was unexpected, as plasma cMyBP-C concentrations were reduced in cats with chronic, treated clinical HCM compared with Sub-C and healthy cats. This finding contrasts with the increased circulating cMyBP-C and cardiac troponin concentrations typically observed during acute cardiomyocyte injury [30,32,33,56,57], suggesting that cMyBP-C may exhibit distinct biomarker patterns according to disease stage and clinical status.

One possible explanation is that chronic myocardial remodeling associated with HCM may alter cMyBP-C release from cardiac tissue, contributing to the reduced plasma concentrations observed in cats with chronic, treated clinical HCM. Previous human studies have shown that myocardial tissue carrying *MYBPC3*-truncating mutations exhibits reduced mutant mRNA expression and an overall decrease of approximately 30% in total full-length cMyBP-C protein levels [58]. Similarly, reduced myocardial cMyBP-C expression has been reported in HCM patients, particularly those with *MYBPC3* mutations [59,60,61]. Although *MYBPC3* mutation status was not evaluated in this study, previous reports indicate that approximately 34–40% of cats with HCM carry *MYBPC3* gene variants, particularly in certain breeds [4,10,13]. The predominance of Scottish Fold and Persian cats and the absence of *MYBPC3* genotyping may therefore limit the generalizability of our findings and preclude assessment of potential breed-associated genetic effects on circulating cMyBP-C concentrations. Accordingly, any association between reduced plasma cMyBP-C and *MYBPC3* haploinsufficiency remains speculative. Importantly, reduced myocardial cMyBP-C expression associated with *MYBPC3* abnormalities should not be interpreted as direct evidence of reduced circulating cMyBP-C concentrations, as the relationship between myocardial protein abundance and circulating biomarker concentrations remains incompletely understood. Nevertheless, reduced myocardial cMyBP-C availability associated with underlying sarcomeric abnormalities may represent one possible explanation for lower circulating cMyBP-C concentrations in some affected cats. However, this hypothesis requires confirmation through integrated genetic analysis, tissue-level assessment, and plasma biomarker evaluation.

Another possible explanation is related to the clinical status and treatment history of the cats included in this study. The clinical HCM cats enrolled in this study represented a chronic, medically stabilized population rather than cats experiencing active MI or acute HF. Therefore, cardiovascular medications, particularly when used concurrently, may have influenced circulating biomarker concentrations through their effects on cardiac loading conditions, hemodynamic status, and myocardial stress or injury. Diuretics may reduce preload and congestion [62,63], ACE inhibitors may modify neurohormonal activation and reduce myocardial fibrosis [64,65], and β-blockers may reduce heart rate and myocardial oxygen demand [66]. In human patients recovering from heart failure events, reductions in plasma cMyBP-C and cardiac troponin concentrations have been reported compared with acute pre-treatment baselines [33,56,67]. Thus, the lower plasma cMyBP-C concentrations observed in the clinical HCM group may not solely reflect intrinsic myocardial or sarcomeric alterations but may also have been influenced by treatment-related effects and clinical stabilization. Given the cross-sectional design of the present study and the absence of pre-treatment biomarker measurements, the relative contributions of underlying disease mechanisms, chronic pharmacotherapy, hemodynamic unloading, and clinical stabilization cannot be distinguished. Survivor bias may also have influenced our findings, as cats with persistent high myocardial injury burden or advanced disease may be underrepresented in a clinically stable cohort. Overall, the mechanism underlying the reduced plasma cMyBP-C concentrations observed in cats with chronic, treated clinical HCM remains unclear. Therefore, these findings should be interpreted cautiously and regarded as hypothesis-generating. Future studies integrating genetic characterization, myocardial tissue analyses, and serial measurements of cMyBP-C and cardiac troponin and longitudinal clinical follow-up are warranted to further elucidate the biological mechanisms underlying circulating cMyBP-C concentrations in feline HCM.

Furthermore, analytical considerations associated with plasma cMyBP-C measurement in feline samples should be acknowledged. The ELISA assay used in this study has been reported by the manufacturer to show no significant cross-reactivity or interference between feline *MYBPC3* and related analogues, supporting its applicability for feline plasma analysis. However, differences in assay calibration and the limited availability of feline-specific biomarker validation data may still influence absolute biomarker quantification. Therefore, further analytical validation in larger feline populations will be important to establish the robustness and clinical applicability of circulating cMyBP-C measurement in cats.

The second major finding of this study was that plasma cMyBP-C demonstrated moderate diagnostic performance for distinguishing cats with HCM from healthy cats, although its overall diagnostic performance (AUC, sensitivity, and specificity) was slightly lower than that of plasma NT-proBNP. Plasma NT-proBNP is currently one of the most widely used cardiac biomarkers for the diagnosis and monitoring of cardiac disease in canines and felines [39,68]. As a biomarker of myocardial wall stress, increased plasma NT-proBNP concentrations reflect the severity of cardiac disease and are widely used to assess congestive processes and, indirectly, cardiac function [39,40]. Our data showed that plasma NT-proBNP concentrations were significantly elevated in clinical HCM cats compared with healthy cats, consistent with previous studies [69,70,71]. Although NT-proBNP concentrations were significantly increased in cats with clinical HCM, the absolute concentrations observed in this study appeared lower than those reported in some previous studies [69,72]. This discrepancy may be related to differences in study populations, disease severity, and clinical status. In particular, the cats with clinical HCM enrolled in the present study represented a chronic, medically stabilized population rather than cats experiencing acute heart failure. Since NT-proBNP reflects myocardial wall stress and hemodynamic burden, lower congestion and greater clinical stability at the time of sampling may have contributed to lower circulating NT-proBNP concentrations [73]. Differences in assay methodology and analytical platforms, together with the lack of independent analytical validation of the research-grade ELISA used in this study, may also influence absolute NT-proBNP values and limit direct comparison with established clinical assays. Therefore, NT-proBNP concentrations should be interpreted within the context of disease stage, treatment status, and assay characteristics, and the absolute values should not be directly extrapolated to established clinical diagnostic thresholds.

Consistent with these principles, combining cMyBP-C and NT-proBNP under the OR rule increased sensitivity, whereas the AND rule increased specificity and positive predictive value, as observed in the present study [74]. However, combining the two biomarkers did not improve overall diagnostic performance compared with NT-proBNP alone. Therefore, based on the current findings, plasma NT-proBNP remains the preferred biomarker for identifying cats with HCM. Although plasma cMyBP-C demonstrated moderate discriminatory ability, its diagnostic performance was insufficient to support its use as an alternative to plasma NT-proBNP. Rather, plasma cMyBP-C should currently be considered a complementary and exploratory biomarker, with potential value for understanding disease biology and biomarker patterns. Its incremental diagnostic value beyond established biomarkers has not yet been demonstrated. Future studies involving larger sample sizes, independent validation cohorts, and longitudinal assessment are warranted to determine whether plasma cMyBP-C provides additional diagnostic or prognostic information beyond established cardiac biomarkers.

The last major finding of this study was that neither plasma NT-proBNP nor plasma cMyBP-C concentrations showed significant correlations with echocardiographic parameters. This lack of association may be related to the relatively small sample size, which may have reduced statistical power and increased the risk of a type II error, heterogeneity in disease severity, variation in underlying myocardial remodeling among individual cats, and the limited ability of echocardiographic measures, particularly wall thickness parameters such as IVSd and LVPWd, to fully reflect myocardial wall stress or active myocardial remodeling. In addition, all clinical HCM cats were sampled after medical treatment and clinical stabilization, which may have reduced the variability in circulating biomarker concentrations and limited the ability to detect relationships with echocardiographic indices. Thus, circulating cMyBP-C and NT-proBNP concentrations may reflect complex interactions among myocardial injury, remodeling, and clinical status rather than structural cardiac changes alone. Future studies incorporating larger cohorts, serial biomarker measurements, and longitudinal echocardiographic assessment are warranted to further clarify these relationships.

Several limitations should also be considered. The cross-sectional design and inclusion of cats with chronic, medically managed HCM limited the evaluation of temporal changes in circulating cMyBP-C and NT-proBNP concentrations and precluded differentiation between disease-related effects and those associated with pharmacotherapy and clinical stabilization. The relatively small sample size and exploratory statistical analyses may also have limited statistical power, warranting confirmation in larger prospective cohorts. In addition, plasma cMyBP-C was measured using a single commercially available ELISA assay without independent analytical validation in feline plasma, and genetic characterization, myocardial tissue assessment, and concurrent cardiac troponin measurements were not performed. Several potential confounding conditions, including systemic hypertension, hyperthyroidism, kidney disease, and other myocardial diseases, were not completely excluded. Conventional echocardiographic parameters may have limited the assessment of cardiac function in cats with concentric hypertrophy, particularly due to the geometric assumptions of Teichholz-derived EF and SV. Future longitudinal studies incorporating comprehensive clinical evaluation, additional indices of systolic and diastolic function, advanced cardiac imaging, and integrated biomarker and genetic analyses are warranted to further clarify the clinical significance of plasma cMyBP-C in feline HCM, including its response to conventional and emerging therapies such as rapamycin.

## 5. Conclusions

In conclusion, this study identified reduced plasma cMyBP-C concentrations in cats with chronic, treated clinical HCM, representing an unexpected finding that may reflect a distinct biomarker pattern compared with acute myocardial injury. However, the mechanisms underlying this observation remain unresolved. Plasma cMyBP-C demonstrated moderate discriminatory performance for identifying cats with HCM but showed lower diagnostic performance than NT-proBNP, which remains the preferred biomarker based on the present findings. Therefore, circulating cMyBP-C should currently be considered an exploratory biomarker with potential complementary value rather than an alternative diagnostic marker. Further studies incorporating genetic characterization, myocardial tissue assessment, and longitudinal biomarker evaluation are warranted to clarify the biological significance of circulating cMyBP-C changes in feline HCM.

## Figures and Tables

**Table 1:** Signalment profile and physical examination of each group.

Parameters	Healthy (*n* = 25)	Sub-C (*n* = 25)	Clinical (*n* = 15)	*p*-Value
Staging ^a^	A = 25	B1 = 12	C = 14	-
		B2 = 13	D = 1	
Sex (female/male) ^b^	13/12	9/16	10/5	0.395
Age (y)	4.00 ± 1.96	5.28 ± 3.46	5.38 ± 3.30	0.216
Body weight (kg)	4.52 ± 1.21	4.56 ± 1.39	4.40 (3.94–5.15)	0.929
BCS (9-point scale)	5.00 (5.00–6.00)	5.00 (5.00–6.00)	5.00 (5.00–6.00)	0.753
Heart rate (bpm)	186.80 ± 12.82	199.60 ± 19.49 *	200.70 ± 31.05 *	0.048
Respiratory rate (tpm)	44.00 (32.00–56.00)	47.10 ± 11.83	48.60 ± 16.03	0.834
Rectal temperature (°F)	101.80 ± 0.45	101.60 ± 0.60	101.10 ± 0.87 *^,†^	0.003

Normally distributed data are expressed as mean ± standard deviation (SD); nonnormally distributed data are expressed as median and interquartile range (IQR). ^a^ Staging classification in this study followed the American College of Veterinary Internal Medicine (ACVIM) consensus statement guidelines for cardiomyopathies in cats [4]. ^b^ Sex distribution was compared using the chi-square test. *p* < 0.05 indicates a statistically significant difference among the three groups. * *p* < 0.05 vs. healthy group, ^†^ *p* < 0.05 vs. subclinical HCM (Sub-C) group; years (y); kilograms (kg); body condition score (BCS); beats per minute (bpm); times per minute (tpm).

**Table 2:** Blood chemistry and hematology profiles of each study group.

Parameters	Healthy (*n* = 25)	Sub-C (*n* = 25)	Clinical (*n* = 15)	*p*-Value
**Blood chemistry**				
BUN (mg/dL)	22.73 ± 4.74	26.20 (23.50–32.15) *	29.30 (26.70–63.60) *	0.0001
Creatinine (mg/dL)	1.45 ± 0.24	1.53 ± 0.27	1.73 ± 0.46 *	0.0270
ALT (U/L)	57.00 (42.50–73.00)	47.00 (38.50–71.50)	46.00 (41.00–77.00)	0.5423
ALP (U/L)	33.00 (24.50–43.50)	30.00 (18.00–44.50)	32.73 ± 17.59	0.7242
Total Protein (g/dL)	8.11 ± 0.77	8.00 (7.25–8.60)	8.44 ± 1.04	0.5234
Albumin (g/dL)	3.58 ± 0.35	3.56 ± 0.37	3.76 ± 0.42	0.2584
**Complete Blood Count**				
Hct (%)	39.20 ± 5.00	37.52 ± 5.39	38.59 ± 4.36	0.4949
Hgb (g/dL)	13.52 ± 1.78	13.45 (11.83–14.38)	14.50 (11.60–14.80)	0.1888
RBC (10^6^/µL)	8.22 ± 0.84	8.08 ± 0.94	8.45 ± 1.20	0.5101
MCV (fL)	47.60 ± 4.11	45.90 (43.35–50.60)	46.01 ± 3.67	0.6274
MCH (pg)	16.42 ± 1.08	15.82 ± 1.20	15.68 ± 0.73	0.0641
MCHC (g/dL)	34.80 (33.85–35.25)	33.78 ± 1.05	34.18 ± 1.71	0.0845
WBC (10^3^/µL)	10.93 ± 2.60	8.22 (7.26–12.17)	11.02 ± 2.02	0.1146
Neu (seg) (10^3^/µL)	6.72 ± 2.08	4.87 (3.31–6.76)	6.77 ± 2.58	0.0637
Lym (10^3^/µL)	3.14 ± 1.13	2.69 ± 1.27	3.19 ± 1.82	0.4052
Mon (10^3^/µL)	0.24 ± 0.12	0.23 ± 0.12	0.26 ± 0.12	0.7928
Eos (10^3^/µL)	0.72 ± 0.35	0.51 ± 0.25	0.39 (0.29–0.87)	0.0612
Bas (10^3^/µL)	0.010 (0.000–0.010)	0.000 (0.000–0.010)	0.010 (0.008–0.125)	0.2381
PLT (10^3^/µL)	245.7 ± 82.0	246.0 ± 66.9	210.0 (163.0–240.0)	0.2099

Normally distributed data are expressed as mean ± standard deviation (SD); nonnormally distributed data are expressed as median and interquartile range (IQR). *p* < 0.05 indicates a statistically significant difference among the three groups. * *p* < 0.05 vs. healthy group. blood urea nitrogen (BUN); alanine aminotransferase (ALT); alkaline phosphatase (ALP); total protein (TP); albumin (ALB); hematocrit (Hct); hemoglobin (Hgb); red blood cell (RBC); mean corpuscular volume (MCV); mean corpuscular hemoglobin (MCH); mean corpuscular hemoglobin concentration (MCHC); white blood cell (WBC); segmented neutrophil (Neu); lymphocyte (Lym); monocyte (Mon); eosinophil (Eos); basophil (Bas); platelet (PLT).

**Table 3:** Thoracic radiographic and echocardiographic findings of each study group.

Parameters	Healthy (*n* = 25)	Sub-C (*n* = 25)	Clinical (*n* = 15)	*p*-Value
**Thoracic radiography**				
VHS	7.77 ± 0.20	7.95 ± 0.52	9.027 ± 1.50 *^,†^	<0.0001
VLAS	1.80 (1.55–1.95)	1.70 (1.50–1.90)	2.05 ± 0.20 *^,†^	0.0010
M-VLAS	2.00 ± 0.27	2.12 ± 0.27	2.54 ± 0.32 *^,†^	<0.0001
RLAD	1.05 (1.00–1.10)	1.20 ± 0.15 *	1.40 (1.28–1.75) *^,†^	<0.0001
**Echocardiographic examination**				
Ao diameter (mm)	7.96 ± 0.11	7.64 ± 0.10	7.23 ± 0.14	0.1522
LA diameter (mm)	11.56 ± 0.15	12.77 ± 0.17	15.00 (13.33–21.30) *^,†^	<0.0001
LA/Ao ratio	1.44 (1.32–1.49)	1.73 ± 0.28 *	2.49 ± 0.85 *^,†^	<0.0001
IVSd (mm)	4.30 ± 0.08	5.86 ± 0.09 *	6.40 ± 0.10 *	<0.0001
LVIDd (mm)	15.64 ± 0.15	14.34 ± 0.25	13.59 ± 0.30 *	0.0216
LVPWd (mm)	4.57 ± 0.07	5.70 ± 0.10 *	7.37 ± 0.21 *^,†^	<0.0001
IVSs (mm)	6.67 ± 0.09	8.00 ± 0.11 *	9.30 ± 0.15 *^,†^	<0.0001
LVIDs (mm)	7.14 ± 0.16	5.93 (5.14–6.96)	6.27 ± 0.24	0.0946
LVPWs (mm)	7.08 ± 0.09	7.83 ± 0.10	9.18 ± 0.16 *^,†^	<0.0001
FS (%)	52.94 ± 8.14	56.02 ± 7.85	51.00 ± 11.47	0.2100
EF (%)	85.22 ± 6.43	89.43 (84.79–90.64)	83.78 ± 9.67	0.1674
SV (mL)	5.33 ± 0.98	4.88 ± 1.55	4.27 ± 1.54	0.0752
CO (mL/min)	1101 ± 262.3	933.5 ± 310.5	899.5 ± 326.9	0.0740
MV E velocity (cm/s) ^a^	79.98 ± 11.89	79.53 ± 23.31	105 ± 33.99 *^,†^	0.0044
MV E/A ratio ^a^	1.22 ± 0.13	1.10 ± 0.30	1.33 (1.17–1.82) ^†^	0.0242

^a^ E/A ratio and E velocity were excluded in cats with E–A wave fusion (1 healthy cat, 2 Sub-C cats, and 3 clinical cats). Normally distributed data are expressed as mean ± standard deviation (SD); nonnormally distributed data are expressed as median and interquartile range (IQR). *p* < 0.05 indicates a statistically significant difference among the three groups. * *p* < 0.05 vs. healthy group, ^†^ *p* < 0.05 vs. Subclinical HCM (Sub-C) group. Vertebral heart score (VHS); vertebral left atrial score (VLAS); modified vertebral left atrial score (M-VLAS); radiographic left atrial dimension (RLAD); aortic root (Ao); left atrium (LA); left atrial-to-aortic ratio (LA/Ao); left ventricular posterior wall thickness at end-diastole (LVPWd); left ventricular internal dimension at end-diastole (LVIDd); interventricular septal thickness at end-diastole (IVSd); left ventricular posterior wall thickness at end-systole (LVPWs); left ventricular internal dimension at end-systole (LVIDs); interventricular septal thickness at end-systole (IVSs); fractional shortening (FS); ejection fraction (EF); stroke volume (SV); cardiac output (CO); peak velocity blood flow from left ventricular relaxation in early diastole (MV E velocity); ratio of peak velocity blood flow from left ventricular relaxation in early diastole to peak velocity flow in late diastole caused by atrial contraction (MV E/A).

**Table 4:** Diagnostic performance of plasma biomarkers for identifying hypertrophic cardiomyopathy in cats.

Parameters	Sensitivity (%)	Specificity (%)	PPV	NPV	Accuracy (%)
One test					
cMyBP-C	76.19	65.38	78.05	62.96	72.06
NT-proBNP	88.89	58.33	76.19	77.78	76.67
Parallel testing (cMyBP-C + NT-proBNP)					
AND	64.71	90.91	91.67	62.25	75.00
OR	97.06	31.82	68.75	87.50	71.43
Serial testing (start with cMyBP-C)					
AND	48.65	92.00	90.00	54.76	66.13
OR	94.87	30.43	69.81	77.78	70.97
Serial testing (start with NT-proBNP)					
AND	64.71	91.30	91.67	63.64	75.44
OR	97.22	30.43	68.63	87.50	71.19

Data were obtained from 40 cats with hypertrophic cardiomyopathy (HCM) and 25 healthy cats. The “OR” rule yields a positive diagnosis if at least one test is positive. The “AND” rule yields a positive diagnosis only if both tests are positive. Positive predictive value (PPV); negative predictive value (NPV).

**Table 5:** Correlation between plasma cardiac biomarkers and echocardiographic parameters in cats with hypertrophic cardiomyopathy (HCM) (*n* = 40).

Parameters	cMyBP-C		NT-proBNP	
	r/r_s_	*p*-Value	r/r_s_	*p*-Value
LA/Ao ratio	0072_s_ = −0.13	0.220	r_s_ = 0.22	0.201
IVSd (cm)	r = −0.008	0.190	r = 0.138	0.422
LVIDd (cm)	r = 0.001	0.397	r = −0.081	0.639
LVPWd (cm)	r_s_ = −0.34	0.445	r_s_ = −0.09	0.594
LVIDs (cm)	r = −0.102	0.963	r = −0.155	0.383
EF (%)	r_s_ = 0.25	0.148	r_s_ = 0.20	0.238
FS (%)	r = 0.170	0.389	r = 0.023	0.893
SV (mL)	r = 0.030	0.275	r = −0.144	0.415
CO (mL/min)	r = 0.120	0.376	r = −0.147	0.408
MV E velocity (cm/s) ^a^	r = −0.156	0.625	r = 0.342	0.064
MV E/A ratio ^a^	r_s_ = −0.06	0.770	r_s_ = 0.15	0.430

^a^ The E/A ratio and E velocity were excluded in cats with E–A wave fusion (2 Sub-C cats and 3 clinical cats). Data are presented as correlation coefficients. N-terminal pro-B-type natriuretic peptide (NT-proBNP); cardiac myosin-binding protein C (cMyBP-C); Pearson correlation coefficient (r); Spearman’s rank correlation coefficient (r_s_); aortic root (Ao); left atrium (LA); left atrial-to-aortic ratio (LA/Ao); left ventricular posterior wall thickness at end-diastole (LVPWd); left ventricular internal dimension at end-diastole (LVIDd); interventricular septal thickness at end-diastole (IVSd); left ventricular posterior wall thickness at end-systole (LVPWs); left ventricular internal dimension at end-systole (LVIDs); interventricular septal thickness at end-systole (IVSs); fractional shortening (FS); ejection fraction (EF); stroke volume (SV); cardiac output (CO); peak velocity blood flow from left ventricular relaxation in early diastole (MV E velocity); ratio of peak velocity blood flow from left ventricular relaxation in early diastole to peak velocity flow in late diastole caused by atrial contraction (MV E/A).

## Data Availability

The original contributions presented in this study are included in the article/Supplementary Material. Further inquiries can be directed to the corresponding author.

## Supplementary Materials

The following supporting information can be downloaded at https://www.mdpi.com/article/10.3390/ani16193084/s1, Table S1. Summary of cardiovascular medications in cats with hypertrophic cardiomyopathy (*n* = 40).

## Abbreviations

HCM	Hypertrophic cardiomyopathy
LV	Left ventricular
cMyBP-C	Cardiac myosin-binding protein C
HF	Heart failure
ATE	Arterial thromboembolism
MI	Myocardial infarction
NT-proBNP	N-terminal pro-B-type natriuretic peptide
ACVIM	American College of Veterinary Internal Medicine
Sub-C	Subclinical HCM
ECG	Electrocardiography
CBC	Complete blood count
BC	Blood chemistry
LA	Left atrium
VHS	Vertebral heart score
VLAS	Vertebral left atrial score
M-VLAS	Modified vertebral left atrial score
RLAD	Radiographic left atrial dimension
Ao	Aortic root
LA/Ao	LA-to-aortic ratio
LVPWd	Left ventricular posterior wall during diastole
LVIDd	LV internal dimension at end-diastole
IVSd	Interventricular septum during diastole
LVPWs	Left ventricular posterior wall during systole
LVIDs	LV internal dimension at end-systole
IVSs	Interventricular septum during systole
FS	Fractional shortening
EF	Ejection fraction
SV	Stroke volume
CO	Cardiac output
MV E peak velocity	Peak velocity blood flow from LV relaxation in early diastole
MV E/A	Ratio of peak velocity blood flow from left ventricular relaxation in early diastole to peak velocity flow in late diastole caused by atrial contraction
ELISA	Enzyme-linked immunosorbent assay
OD	Optical density
SD	Standard deviation
IQR	Interquartile range
ROC	Receiver operating characteristic
PPV	Positive predictive value
NPV	Negative predictive value
ACEis	Angiotensin-converting enzyme inhibitors
AUC	Area under the curve
