# Supplementary material for: Reduced Plasma Cardiac Myosin-Binding Protein-C Concentrations in Cats with Hypertrophic Cardiomyopathy: Evidence from Chronic, Treated Disease
Source: Animals (Basel). 2026 Sep 30;16(19):3084. doi: 10.3390/ani16193084 (PMC13663380; doi:10.3390/ani16193084)
Supplement: Supplementary file 1 [file animals-16-03084-s001.zip › animals-4501744-supplementary.pdf]

Table S1. Summary of cardiovascular medications in cats with hypertrophic cardiomyopathy (*n* = 40).

Medicine	Dose range	Sub-C	Dose range	Clinical
**Individual medications**				
ACEis		3 (12.0)		6 (40.0)
• Benazepril<br>• Ramipril	• 0.25–0.5 mg/kg, q 24 h<br>• 0.125–0.25 mg/kg, q 24 h	• 2<br>• 1	• 0.25–0.5 mg/kg, q 24 h<br>• 0.125–0.25 mg/kg, q 24 h	• 4<br>• 2
Antiplatelet agent				
• Clopidogrel	• 18.75 mg/cat, q 24 h	3 (12.0)	• 18.75 mg/cat, q 24 h	15 (100)
β-blocker				
• Atenolol	• 6.25–12.5 mg/cat, q 24 h	2 (8.0)	• 6.25–12.5 mg/cat, q 24 h	4 (26.7)
CCBs				
• Diltiazem	• 1.75–2.5 mg/kg, q 12 h	2 (8.0)	• 1.75–2.5 mg/kg, q 8–12 h	2 (13.3)
Loop diuretic				
• Furosemide	–	0 (0.0)	• 1–4 mg/kg, q 8–12 h	10 (66.7)
Positive inotrope				
• Pimobendan	–	0 (0.0)	• 0.25–0.3 mg/kg, q 12 h	3 (20.0)
Aldosterone antagonist				
• Spironolactone	–	0 (0.0)	• 1–4 mg/kg, q 12–24 h	3 (20.0)
**Numbers of medications per cat**				
	**Sub-C**		**Clinical**	
0 medication	20 (80.0)		0 (0.0)	
1 medication	1 (4.0)		3 (20.0)	
2 medications	3 (12.0)		3 (20.0)	
3 medications	1 (4.0)		4 (26.7)	
4 medications	0 (0.0)		3 (20.0)	
≥5 medications	0 (0.0)		2 (13.3)	
**Overall treatment duration (months)**[a]				
	9 ± 6		1 (1-12)	

Data are presented as *n* (%), mean ± standard deviation (SD), or median and interquartile range (IQR), as appropriate. Percentages were calculated within each study group. Overall treatment duration was compared between the subclinical (Sub-C) and clinical hypertrophic cardiomyopathy (HCM) groups using the Mann–Whitney U test, with no significant

difference observed between groups ($p = 0.1127$). [a] Overall treatment duration was defined as the period from initiation of cardiovascular treatment to blood sampling. The duration of individual medications was not consistently available. In cats requiring treatment adjustment, the most recent medication regimen had remained stable for at least 1 month before blood sampling. Medical therapy was not included as a covariate in the statistical analyses because of the small number of cats within individual treatment subgroups and the frequent use of multiple cardiovascular medications concurrently, which precluded reliable assessment of drug-specific effects. Angiotensin-converting enzyme inhibitor (ACEis); calcium channel blocker (CCBs); every 8 h (q 8 h); every 12 h (q 12 h); every 24 h (q 24 h).
